# Low Muscle Mass in Patients Receiving Hemodialysis: Correlations with Vascular Calcification and Vascular Access Failure

**DOI:** 10.3390/jcm10163698

**Published:** 2021-08-20

**Authors:** Seok-hyung Kim, Gwangho Choi, Youngjin Song, Hojung Yoon, Hae Min Jeong, Jae Eon Gu, Miyeun Han, Jongho Heo, Jeong-Ju Yoo, Jong-woo Yoon, Hyunsuk Kim

**Affiliations:** 1 Department of Internal Medicine, Chuncheon Sacred Heart Hospital, Chuncheon 24253, Korea; thrupy7@hallym.or.kr (S.-h.K.); sindowhikaru@hallym.or.kr (G.C.); songyj141@hallym.or.kr (Y.S.); memory493@hallym.or.kr (H.Y.); cromnjeong@hallym.or.kr (H.M.J.); ggucci99@hanmail.net (J.E.G.); yoonjw@hallym.or.kr (J.-w.Y.); 2 Department of Internal Medicine, Hangang Sacred Heart Hospital, Seoul 07247, Korea; myeun81@hanmail.net; 3 National Assembly Futures Institute, Seoul 07233, Korea; jjongho77@gmail.com; 4 Department of Internal Medicine, Soonchunhyang University Hospital Bucheon, Bucheon 14584, Korea; puby17@schmc.ac.kr

**Keywords:** hemodialysis, vascular calcification, sarcopenia, vascular access failure

## Abstract

**Background:** Sarcopenia involves an age-related decline in skeletal muscle mass with functional disability or low muscle strength. Vascular calcification (VC) occurs commonly in patients with chronic kidney disease, in whom it is associated with cardiovascular disease. We aimed to investigate the correlations of low muscle mass with the quantified vascular calcification score (VCS) of the arm of vascular access, as well as whether low muscle mass is associated with the incidence of vascular access failure. **Methods:** The VCS was measured on non-contrast, arm computed tomography using the Agatston method. The lower muscle mass (LMM) group comprised subjects whose skeletal muscle mass of the lower extremities, as measured using bioelectrical impedance, was lower than the median. Higher VC was defined as a score of 500 or above, corresponding to the highest 40% of VCS. The relationship between LMM and VC was explored using univariate and multivariate logistic regression analyses. **Results:** Seventy-five patients were included, of whom forty-two (56.0%) were men. The median age was 64 years (interquartile range 58–72 years). Of the 75 patients, 73 satisfied the diagnostic criteria for sarcopenia. The median hemodialysis vintage was 49.4 months (range 32.1–99.2 months). No significant differences were found between the non-LMM and LMM groups in sex, end-stage renal disease etiology, and type of vascular access, although the LMM group showed significantly older age and hemodialysis vintage. LMM presented a significant association with VC (hazard ratio (HR) 3.562; 95% CI, 1.341–9.463; *p* = 0.011). Upon adjustment for hemodialysis vintage, diabetes, and systolic blood pressure, LMM demonstrated an independent association with VC (HR, 10.415; 95% CI, 2.357–46.024; *p* = 0.002). The risk of vascular access failure was higher in the LMM group (HR, 3.652; 95%, CI 1.135–11.749; *p* = 0.03). VC was a full mediator in the relationship of LMM with recurrent vascular access failure. **Conclusions:** We quantified LMM via bioimpedance analysis and found a heretofore-unreported association between LMM and vascular access failure. LMM increases the risk of VC and has the potential to predict vascular access failure.

## 1. Introduction

Sarcopenia is a term of Greek origin referring to physical poverty or insufficiency [1]. It was first used in the 1980s to describe a phenomenon involving a decrease in lean body mass and alterations in physiological function affecting mortality and morbidity [2]. More recently, the European Working Group on Sarcopenia in Older People (EWGSOP) defined sarcopenia as a syndrome involving the age-related loss of skeletal muscle mass and strength that poses a risk of adverse outcomes, including poor quality of life, physical disability, and death [3]. Sarcopenia is suspected in the presence of low muscle strength, and its diagnosis is confirmed if low muscle quantity or quality is found. Sarcopenia is considered to be severe in patients with low muscle strength, low muscle quantity or quality, and low physical performance [4]. Although these general criteria apply to all people, regardless of ethnicity, Asian people require special consideration regarding the diagnosis of sarcopenia because of anthropometric differences compared to western populations [5]. Therefore, the Asian Working Group for Sarcopenia (AWGS) published an updated diagnostic algorithm and criteria drawing upon Asian data. In these criteria, the AWGS followed the EWGSOP diagnostic approach, but provided different cutoff values from the EWGSOP criteria. The AWGS proposed the following cutoffs for diagnosing sarcopenia: low muscle strength is defined as a handgrip strength <18 kg for women and <28 kg for men; the cutoff for physical performance is a 6 min walk test <1.0 m/s, a time-time chair stand test ≥12 s, or a Short Physical Performance Battery score ≤9; and low muscle mass is defined as a height-adjusted muscle mass <5.7 kg/m^2^ in women and <7.0 kg/m^2^ in men using dual-energy X-ray absorptiometry (DXA), or <5.4 kg/m^2^ in women and <7.0 kg/m^2^ in men using bioimpedance [6].

Cardiovascular events constitute the most common cause of death in CKD patients [7]. For this reason, the clinical assessment of risk factors for cardiovascular mortality is of the utmost importance in patients with CKD. As a cardiovascular risk factor, vascular calcification (VC) is a substantial contributor to cardiovascular morbidity and mortality in patients with end-stage renal disease (ESRD) [8]. A previous study showed that VC in ESRD patients was associated with an elevated risk of major adverse cardiovascular and cerebrovascular events (MACCEs). In that study, after quantifying the VC of the access arm using computed tomography (CT), it was shown that the high-VC group had a higher risk for both MACCEs and access failure [9]. Moreover, according to a recent study, noncoronary VC was correlated with sarcopenia [10,11]. It was suggested that the pathogenesis of sarcopenia and VC involves several shared mechanisms [12]. However, few published studies have explored the correlation between VC at the vascular access site and sarcopenia, which is also a strong predictor of cardiovascular risk [13]. Likewise, little research has focused on the clinical importance of VC at the vascular access site in patients with ESRD.

To address the above-described gap in the research, we quantified VC in patients with ESRD using the Agatston method and investigated the correlations of VC with sarcopenia and vascular access failure. We also aimed to clarify whether low muscle mass was associated with access failure in the context of VC at the vascular access site.

## 2. Materials and Methods

### 2.1. Patients

To estimate the sample size, we used results from a previous clinical study of the association between VC and sarcopenia [14]. Assuming an odds ratio (OR) of 3.78 for the prevalence of VC in the sarcopenia group, at least 73 patients were needed (α < 0.05 and power of 95%). The sample size calculation was performed using a power analysis program (G*power, http://gpower.hhu.de/ accessed on 3 June 2019). This cross-sectional study included 75 patients who received hemodialysis on an outpatient basis for at least 3 months. Study patients were enrolled from January 2019 to May 2019 at a single center. Patients with an arteriovenous fistula or arteriovenous graft in the forearm or upper arm, but not those with a hemodialysis catheter, were included. This study excluded patients who (1) were unable to move without walking aids; (2) had an active malignancy; (3) had a history of chronic heart failure; or (4) had a history or current diagnosis of a systemic disease affecting the prevalence of sarcopenia. CT scans were used to determine the vascular calcification score (VCS). Ferritin, albumin, cholesterol, hemoglobin, chronic kidney disease–mineral and bone disorder (CKD-MBD) parameters, and single pool (sp) Kt/V were recorded. A retrospective review of patients’ medical records from January 1985 to May 2019 was conducted to collect information on patients’ medical history, including blood pressure, comorbidities, ESRD etiology, hemodialysis vintage, details of vascular access, ultrafiltration volume, height, weight, and dry weight. There were 43 men and 33 women. One patient with a femoral arteriovenous graft was excluded; therefore, the final analysis included 75 patients. Vascular interventions, such as surgical thrombectomy or percutaneous transluminal angioplasty (PTA), were carried out in patients who developed access dysfunction, such as acute thrombotic occlusion, delayed hemostasis at the puncture site, and elevated venous pressure. All procedures were conducted in accordance with the relevant ethical standards of institutional and national research committees and with the 1964 Helsinki Declaration and its later amendments or comparable ethical standards. The study protocol received approval from the institutional review board (IRB No. Hallym 2019-03-015).

### 2.2. Measurement of the Muscle Mass of Both Lower Extremities Using Bioimpedance Analysis

Bioimpedance analysis (BIA) measurements were obtained at dialysis sessions held in the middle of the week. Patients were told to consume meals 2 h before dialysis to prevent interference from the meal, and they were not permitted to eat while dialysis was being performed. After dialysis, patients were instructed to lie flat on the bed after their post-hemodialysis weight was measured. Thereafter, patients rested for 10 min in the lying position, and then BIA measurements were recorded using an S10 device (InBody, Seoul, South Korea). Electrodes were attached to both the feet (lateral and medial sides) and the hands (middle finger and thumb). BIA estimates muscle mass indirectly using electrical conductivity. The muscle quantity of the upper extremities can be influenced by the presence of the hemodialysis shunt in the arm. However, the muscle mass of the lower extremities is not directly influenced by the hemodialysis shunt. Thus, when quantifying muscle mass, leg skeletal muscle (LSM) was calculated as the sum of the skeletal muscle mass of both legs. Since muscle mass is fundamentally correlated with body size, LSM was adjusted using height squared (LSM/height^2^). The height-corrected LSM was termed the leg skeletal muscle index (LSMI). The lower muscle mass (LMM) group was defined as subjects with a lower LSMI than the median for each sex as an operational definition of sarcopenia.

### 2.3. Measurement of the Vascular Calcification Score

CT scans extending from the chest to the pelvis were obtained (120 kVp, 135 mAs, and slice thickness 3 mm). The radiation dose was calculated using the median direct dose profile integral (DPI). A value of approximately 500 DPI was obtained, corresponding to a similar or lower amount of radiation than the dose of a non-contrast CT scan of the same area. The Agatston method was used to calculate the VCS by multiplying the area of each calcified lesion by a weighting factor derived from Hounsfield units. The 75 patients were classified based on their VCS score (≤500 or >500). In a previous study, a cutoff value of 500 predicted a high risk of MACCEs. A detailed description of the methods and rationale was presented in a previous study [9].

### 2.4. Statistical Analysis

Data are presented as median and interquartile range (IQR; 25th to 75th percentile) for continuous variables or as frequency (percentage) for categorical variables. The Mann–Whitney U test was used to determine the statistical significance of between-group differences in clinical characteristics for continuous variables, whereas the chi-square test was used for categorical variables. The association between LMM and VC was investigated using univariate and multivariate logistic regression analyses, adjusting for confounders.

Mediation analyses using the Baron and Kenny model were performed to assess the proportion of the total effect on vascular access failure that could be explained by LMM. All analyses were carried out in SPSS version 23.0 (IBM Corp., Armonk, NY, USA).

## 3. Results

### 3.1. Baseline Characteristics of the Subjects

Table 1 presents the subjects’ baseline demographic and clinical characteristics. The participants were divided into two groups according to muscle mass. There were 35 (46.7%) patients with LMM and 40 (53.3%) without LMM. The participants with LMM were significantly older than the participants with higher muscle mass (median [IQR]: 60.5 [51.8, 66.8] years vs. 67.0 [62.5, 76.0] years; *p* = 0.001). No significant differences were found between the two groups in sex, access type, or the prevalence of diabetes. Other clinical markers such as blood pressure, hemoglobin, and uric acid also did not show significant differences.

As related to hemodialysis, dry weight was significantly lower in participants with LMM (median [IQR]: 61.3 [54.0, 71.4] kg vs. 56.5 [47.8, 60.8] kg; *p* = 0.036). However, the two groups showed no statistically significant differences in other hemodialysis-related parameters, such as the urea reduction ratio, spKt/V, height, or ultrafiltration volume.

Patients with LMM showed significantly lower 25(OH) vitamin D levels (median [IQR]: 21.9 [15.9, 27.2] ng/mL vs. 17.1 [13.4, 22.8] ng/mL; *p* = 0.027). The levels of other nutritional parameters, such as triglycerides, albumin, and ferritin, did not show significant differences. The remaining variables related to CKD-MBD were also not significantly different between the two groups.

### 3.2. Associations of Vascular Calcification with Risk Factors Including Lower Muscle Mass

Univariate logistic regression analysis was conducted to explore the associations of covariates with VC in order to identify risk factors for VC. The VC group was defined as patients with a VCS > 500. Among the variables related to hemodialysis, the presence of an arteriovenous graft and longer hemodialysis duration were significantly associated with VC (vascular access: OR 5.460 (95% CI 1.312–22.722), *p* = 0.020, HD vintage: OR 1.016 (95% CI 1.006–1.027), *p* = 0.003). Among the anthropometric measurements, low BMI showed a significant relationship with VC (OR 0.841 (95% CI 0.174–0.993], *p* = 0.041). Nutritional parameters were not associated with VC, except albumin (OR 0.139 (95% CI 0.030–0.651], *p* = 0.012). Finally, LMM presented a significant association with VC (OR 3.562 (95% CI 1.341–9.463), *p* = 0.011) (Table 2).

**Figure 1:**
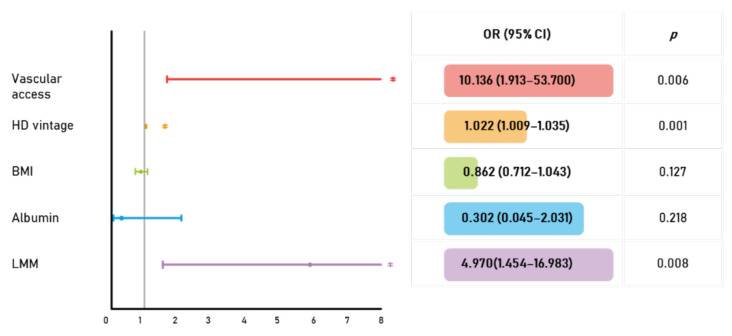
Multivariate logistic regression analysis of risk factors of vascular calcification (VC)**:** Forest plot (left) and odds ratios for the association between LMM and VC after adjustment for multiple confounders. The presence of an arteriovenous graft, longer hemodialysis duration, and low muscle mass were independent predictors of VC. * *p* < 0.05. AVG, arteriovenous graft; AVF, arteriovenous fistula; HD, hemodialysis; BMI, body mass index; LMM, low muscle mass; OR, odds ratio; CI, confidence interval.

Multivariate analysis was carried out to identify independent predictors of VC. The presence of an arteriovenous graft (versus arteriovenous fistula), longer hemodialysis duration, and low muscle mass showed independent associations with VC (vascular access: OR 10.136 (95% CI 1.913–53.700), *p* = 0.006, HD vintage: OR 1.022 (95% CI 1.009–1.035), *p* = 0.001, LMM: OR 4.970 (95% CI 1.454–16.983), *p* = 0.008) (Figure 1).

### 3.3. Associations among Vascular Calcification, Muscle Mass, and the Prevalence of Vascular Access Failure

In the group with higher VCS, 21 (72.4%) patients received interventions (e.g., surgical thrombectomy, PTA, or revision), whereas only nine (19.6%) of the patients in the group with lower VCS experienced interventions. In the group with higher VCS, there were 15 (51.7%) subjects who received two or more interventions. However, only two (4.3%) subjects underwent at least two interventions in the group with lower VCS. In this study, the requirement for two or more interventions was defined as recurrent vascular access failure. The higher-VCS group also had a higher number of annual interventions (lower VCS: 0.0 [IQR 0.0, 0.0] vs. higher VCS: 0.2 [0, 0.6]; *p* < 0.001).

LMM was also correlated with vascular access failure. In the LMM group, 17 (48.6%) subjects underwent interventions including PTA or surgical thrombectomy or revision. In the non-LMM group, 13 (32.5%) subjects received interventions, but this difference from the LMM group did not reach statistical significance. The LMM group contained significantly more subjects who experienced recurrent vascular access failure than the non-LMM group (non-LMM: 5 (12.5%) vs. LMM: 12 (34.3%); *p* = 0.025). The LMM group also had a higher number of annual interventions (non-LMM: 0.00 [IQR 0.00, 0.18] vs. LMM: 0.00 [IQR 0.00, 0.30]; *p* = 0.042). (Table 3). Although there was no difference in the access type between the LMM and non-LMM groups, patients with AVG had a higher incidence of access failure (72.7% vs. 27.3%, *p* = 0.016) and recurrent access failure (54.5% vs. 17.2%, *p* = 0.006) than patients with AVF.

**Figure 2:**
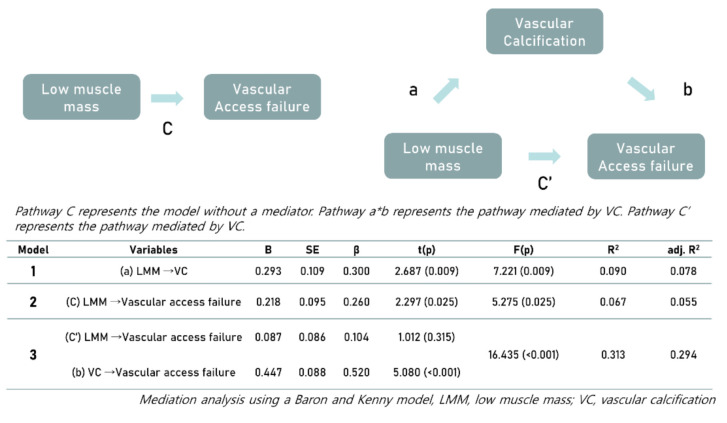
VC as a mediator of the association between LMM and recurrent vascular access failure. Mediation analysis of the association between VC and recurrent vascular access failure in which VC was considered as a mediator. Pathway C represents the model without a mediator. Pathway a*b represents the pathway mediated by VC. Pathway C’ represents the pathway not mediated by VC. Consequently, LMM is associated with vascular access failure, which mediates VC. Mediation analysis using the Baron and Kenny model. LMM, low muscle mass; VC, vascular calcification.

We assessed the potential role of VC as a mediator in the relationship between LMM and recurrent vascular failure. LMM was independently associated with VC or recurrent vascular failure. The mediation analysis showed no significant association between LMM and recurrent vascular failure when VC did not mediate the relationship (Figure 2). When VC participated as a mediator, LMM and vascular access failure lost their association, and VC still mediated vascular access failure. This means that VC acts as a full mediator and is essential for LMM to facilitate vascular access failure. Consequently, LMM is associated with vascular access failure, which mediates VC.

## 4. Discussion

In this study, the LMM group classified by the median value of LSMI showed a significant association with VC. This significant association persisted after adjusting for several possible confounders. As expected, VC was significantly associated with recurrent vascular access failure requiring interventions, including PTA or surgical thrombectomy. Low muscle mass was also associated with recurrent vascular failure. VC functioned as a full mediator in the relationship between muscle mass and recurrent vascular failure. This means that VC was an essential factor linking LMM with recurrent vascular access failure.

Several age-related changes contribute to sarcopenia [15]. Aging is accompanied by a diminished expression of hormones, such as cortisol or myostatin, that upregulate protein synthesis, as well as by increased expression of inflammatory and endocrine factors that increase protein degradation and thereby cause a negative protein balance [16,17]. In this study, all but two subjects met the criteria for low muscle mass suggested by the AWGS. In patients with CKD, sarcopenia develops more intensively and at a younger age than would otherwise be expected [18]. The mechanisms predisposing patients with CKD to the development of sarcopenia are associated with phenomena related to kidney disease (e.g., hormonal changes, metabolic acidosis, nutritional deficiencies, anemia, vitamin D deficiency, CKD-MBD factors, insulin resistance, and proteinuria) and the developing inflammatory process triggered by proinflammatory cytokines and oxidative stress, particularly in hemodialysis patients [19,20].

Bones are the main reservoir of total body calcium. Calcium salts are also stored in tissues and organs outside the bones, which is commonly called extraskeletal calcification [21]. Some types of cells with unregulated osteogenic and chondrogenic potential cause calcification, especially in the arterial wall [22]. In the 19th century, VC was regarded as the presence of bone tissue within atherosclerotic arteries [23]. However, in the late 20th century, VC was found to involve chondrocytes and osteoblast-like cells, which form from the de-differentiation of vascular smooth muscle cells. Although chondrocytes and osteoblasts are different cell types, they participate in a similar mineralization mechanism and have similar effects on gene expression involving VC. Major regulators are expressed in atherosclerotic plaques, such as bone morphogenetic protein-2, osteoprotegerin, matrix γ-carboxyglutamic acid protein, and osteopontin [24]. The mechanisms of VC in CKD patients are distinct from those of VC in the normal population or patients affected by other diseases [25]. In CKD patients, the above-mentioned de-differentiation of vascular smooth muscle cells into osteoblast-like cells or chondrocytes is promoted by various risk factors [26]. Furthermore, CKD–MBD patients characteristically have high phosphorus and calcium levels [27], and CKD patients are thought to have diminished expression levels of signaling molecules that exert an inhibitory effect on VC [28]. For these reasons, CKD patients have a higher risk of VC than in the general population, as reflected by a 2–5 times higher prevalence of VC in CKD patients than in age-matched patients without CKD [29].

A main cause of VC is oxidative stress and inflammation leading to a loss of the balance between endothelial damage and repair [30]. Hormonal dysregulation (e.g., insulin resistance) and microcalcification induced by oxidative stress and inflammation may mechanically contribute to skeletal muscle loss due to reduced nutrients, diminished capillary microcirculation, and reduced oxygen transfer, thereby contributing to impairments in muscle protein synthesis [31] and higher levels of protein degradation [32]. Aging-associated decreases in other hormone levels might exert harmful effects on muscle metabolism by diminishing the flow of blood within skeletal muscles [33]. Insulin-like growth factor 1 (IGF-1) was reported to protect against aging-associated losses of muscle mass and strength [34]. In skeletal muscle, IGF-1 muscle activates the PI3K/Akt pathway [35]. Insulin-stimulated protein synthesis requires the activation of Akt [36]. Through this mechanism, IGF-1 stimulates protein synthesis and inhibits muscle breakdown, and it is therefore logical that the aging-associated decline in IGF-1 levels has a negative effect on skeletal muscle.

VC induces vascular access failure through several molecular mechanisms [37]. The risk of arteriovenous fistula interventions is associated with VC markers, such as fetuin-A, osteopontin, osteoprotegerin, and bone morphogenetic protein-7 [38]. However, to the best of our knowledge, no reports have shown an association between low muscle mass and recurrent vascular access failure. A major strength of this study is that it demonstrates the influence of VC as a mediator, as well as presenting an association between low muscle mass and recurrent vascular access failure.

However, there are several limitations to this study. First, the cross-sectional nature of the study design precludes an interpretation in terms of causal relationships. Second, bioimpedance was used to estimate skeletal muscle mass. Bioimpedance measurements of muscle mass depend on the relationship between body composition and body water. Thus, bioimpedance can be inaccurate in some conditions that cause hypervolemia, such as CKD or heart failure. However, in patients with CKD, skeletal muscle mass measurements using bioimpedance are as reliable as those obtained using DXA [39]. Moreover, skeletal muscle mass as determined by bioimpedance was found to be reasonably accurate compared with DXA in ESRD patients [40]. In addition, bioimpedance was proposed as the best surrogate method to assess muscle mass consistent with DXA [41]. In our study, BIA was performed at mid-week dialysis sessions to avoid hypovolemia, and patients with definite volume overload were excluded. Third, although muscle strength and performance are essential components of the diagnosis of sarcopenia, they were not measured. Muscle function, as represented by muscle strength and performance, may affect the relationship between skeletal muscle mass and VC described herein. However, a recent study showed a significant direct association between VC and low muscle mass [42].

In conclusion, LMM showed independent relationships with VC and recurrent vascular access failure. VC was found to play a crucial role in the relationship between LMM and recurrent vascular access failure. LMM may be valuable to consider as a risk factor for vascular access failure in the context of VC at the vascular access site.

## Figures and Tables

**Table 1:** Baseline characteristics of study patients classified by muscle mass.

Variables	Non-LMM	LMM	*p*-Value
N (%)	40 (53.3)	35 (46.7)	
Male sex, n (%)	24 (60.0)	18 (51.4)	0.456
Age, years, median [IQR]	60.5 [51.8, 66.8]	67.0 [62.5, 76.0]	0.001
Etiology, n (%)			0.185
Diabetes	17 (42.5)	22 (62.9)	
HTN	14 (35.0)	11 (31.4)	
CGN	1 (2.5)	0	
PKD	1 (2.5)	1 (2.9)	
Others	7 (17.5)	1 (2.9)	
SBP, mmHg, median [IQR]	150.0 [130.0, 167.5]	160 [130.0, 170.0]	0.476
DBP, mmHg, median [IQR]	70.0 [70.0, 80.0]	70 [60, 80]	0.301
Pulse pressure, mmHg, median [IQR]	70 [60, 87.5]	80 [60, 90]	0.057
AVF (vs. AVG), n (%)	37 (92.5)	27 (77.1)	0.061
HD vintage, months, median [IQR]	48.8 [32.2, 108.4]	50.5 [25.7, 101.9]	0.975
UF volume, kg, median [IQR]	2.2 [1.8, 3.1]	1.8 [1.3, 2.8]	0.092
Height, cm, median [IQR]	164.6 [155.2, 171.2]	158 [153.1, 167.3]	0.158
Dry weight, kg, median [IQR]	61.3 [54.0, 71.4]	56.5 [47.8, 60.8]	0.036
BMI, kg/m^2^, median [IQR]	22.7 [20.3, 25.7]	21.5 [20.1, 22.5]	0.124
spKt/V, median [IQR]	1.72 [1.56, 2.03]	2.01 [1.66, 2.24]	0.132
URR, %, median [IQR]	75.8 [73.0, 80.5]	80.6 [74.5, 83.7]	0.111
Hb, g/dL, median [IQR]	10.3 [9.8, 11.1]	10.4 [9.6, 10.9]	0.762
Uric acid, mg/dL, median [IQR]	7.1 [6.2, 8.8]	6.8 [5.9, 7.9]	0.444
Total cholesterol, mg/dL, median [IQR]	123.5 [107.5, 143.5]	126.0 [107.0,147.0]	0.920
LDL-C, mg/dL, median [IQR]	66.0 [50.3, 77.8]	63.0 [46.5, 84.0]	0.766
Triglycerides, mg/dL, median [IQR]	103.5 [70.5, 142.0]	108.0 [85.5, 169.5]	0.413
Albumin, g/dL, median [IQR]	3.9 [3.5,4.0]	3.6 [3.4, 3.9]	0.096
Ferritin, ng/mL, median [IQR]	171.4 [83.8, 213.8]	201.4 [109.3, 300.3]	0.214
CRP, mg/L, median [IQR]	0.1 [0.1, 0.1]	0.1 [0.1, 9.0]	0.003
25(OH) vitamin D, ng/mL, median [IQR]	21.9 [15.9, 27.2]	17.1 [13.4, 22.8]	0.027
Calcium, mg/dL, median [IQR]	8.1 [7.6, 8.6]	8.1 [7.7, 8.5]	0.710
Phosphorus, mg/dL, median [IQR]	5.0 [3.8, 5.9]	4.6 [4.0, 5.6]	0.679
iPTH, pg/mL, median [IQR]	347.0 [146.0, 494.8]	220.0 [134.0, 371.0]	0.206
Magnesium, mg/dL, median [IQR]	2.6 [2.4, 2.9]	2.6 [2.5, 2.8]	0.928

Values are expressed as number (%) or median (interquartile range [IQR], 25th to 75th percentile) of subjects unless noted otherwise. HTN, hypertension; CGN, chronic glomerulonephritis; PKD, polycystic kidney disease; SBP, systolic blood pressure; DBP, diastolic blood pressure; AVF, arteriovenous fistula; AVG, arteriovenous graft; HD, hemodialysis; UF, ultrafiltration; BMI, body mass index; URR, urea reduction rate; Hb, hemoglobin; LDL-C, low-density lipoprotein-cholesterol; CRP, C-reactive protein; iPTH, intact parathyroid hormone.

**Table 2:** Univariate logistic regression analysis to identify factors independently affecting vascular calcification.

Variable	OR (95% CI)	*p*-Value
Sex	1.900 (0.728–0.459)	0.190
Age	0.997 (0.955–1.041)	0.894
Diabetes	1.231 (0.484–3.127)	0.663
SBP	0.992 (0.973–1.011)	0.398
DBP	0.971 (0.934–1.010)	0.971
Pulse pressure	0.992 (0.970–1.015)	0.495
Vascular access	5.460 (1.312–22.722)	0.020
HD vintage	1.016 (1.006–1.027)	0.003
UF volume	1.140 (0.720–1.804)	0.577
Height	0.996 (0.947–1.047)	0.867
Dry weight	0.963 (0.921–1.006)	0.093
BMI	0.842 (0.714–0.993)	0.041
spKt/V	2.250 (0.537–9.426)	0.267
URR	1.047 (0.963–1.138)	0.285
Hb	0.995 (0.636–1.555)	0.981
Uric acid	0.894 (0.685–1.169)	0.414
Total cholesterol	0.987 (0.971–1.003)	0.121
LDL-cholesterol	0.987 (0.969–1.006)	0.188
Triglyceride	0.993 (0.986–1.001)	0.087
Albumin	0.139 (0.030–0.651)	0.012
Ferritin	0.996 (0.992–1.001)	0.095
CRP	0.996 (0.957–1.037)	0.848
25(OH) vitamin D	0.994 (0.944–1.046)	0.805
Calcium	1.115 (0.585–2.123)	0.741
Phosphorus	1.050 (0.762–1.447)	0.766
iPTH	1.001 (0.999–1.003)	0.290
Magnesium	1.660 (0.529–5.208)	0.385
LMM	3.562 (1.341–9.463)	0.011

OR, odds ratio; SBP, systolic blood pressure; DBP, diastolic blood pressure; HD, hemodialysis; UF, ultrafiltration; BMI, body mass index; URR, urea reduction rate; Hb, hemoglobin; LDL-C, low-density lipoprotein-cholesterol; CRP, C-reactive protein; iPTH, intact parathyroid hormone; LMM, lower muscle mass.

**Table 3:** Comparison of the incidence of vascular access failure according to muscle mass.

Variables	Non-LMM	LMM	*p*-Value
Intervention (PTA or surgery), n (%)	13 (32.5)	17 (48.6)	0.156
Recurrent vascular access failure, n (%)	5 (12.5)	12 (34.3)	0.025
Number of interventions/year, median [IQR]	0.00 [0.00, 0.18]	0.00 [0.00, 0.30]	0.042

Values are expressed as number (%) or median (IQR, 25th to 75th percentile). PTA, percutaneous transluminal angioplasty; IQR, interquartile range.

## Data Availability

The datasets of the current study are available from the corresponding author on reasonable request.
